# Case report: Tirzepatide-responsive refractory diabetes mellitus in an adult female with prader–willi syndrome

**DOI:** 10.1097/MD.0000000000050605

**Published:** 2026-09-04

**Authors:** Shuang Li, Junjie Yu, Jilin Wei, Meihua Liang

**Affiliations:** a Department of Endocrinology and Metabolism, Jiangsu Province (Suqian) Hospital, Suqian, China; b Department of Endocrinology and Metabolism, The Second Affiliated Hospital of Harbin Medical University, Harbin, China.

**Keywords:** case report, diabetes mellitus, GIP/GLP-1 receptor agonist, obesity, Prader–Willi syndrome

## Abstract

**Rationale:**

Prader–Willi syndrome (PWS) is a complex genetic disorder that affects multiple systems and results from loss of expression of paternally inherited genes in the 15q11.2–q13 region. It is characterized by severe hypotonia and feeding difficulties during infancy, followed by hyperphagia in later childhood and adolescence, which, without strict control of excessive eating behaviors, ultimately leads to severe obesity associated with type 2 diabetes mellitus.

**Patient concerns:**

We present the case of a woman who was diagnosed with PWS and diabetes mellitus in adulthood. She had a history of hypotonia, developmental delay, intellectual disability, hyperphagia, and morbid obesity since childhood, with gonadal dysplasia emerging during puberty. She presented with long-term poorly controlled blood glucose levels.

**Diagnoses:**

Genetic testing revealed a heterozygous deletion in the 15q11.2–q13.1 region on chromosome 15, and DNA methylation analysis confirmed the diagnosis of PWS.

**Interventions:**

The distinctiveness of this case lies in the patient’s uncontrollable food-seeking behavior combined with PWS-related severe insulin resistance and β-cell dysfunction, which together resulted in refractory diabetes. Initial combination therapy using multiple glucose-lowering agents, including insulin, metformin, chiglitazar sodium, a glucagon-like peptide-1 receptor agonist, and a sodium-glucose cotransporter-2 inhibitor, demonstrated limited efficacy.

**Outcomes:**

Subsequent substitution of the glucagon-like peptide-1 receptor agonist with the dual glucose-dependent insulinotropic polypeptide/glucagon-like peptide-1 (GLP-1) receptor agonist tirzepatide led to notable improvements in appetite suppression, body weight reduction, and glycemic control.

**Lessons:**

This case underscores the importance of considering genetic syndromes in patients with diabetes presenting with early-onset severe obesity and developmental abnormalities and emphasizes the potential therapeutic value of novel antidiabetic agents such as tirzepatide for managing PWS-associated metabolic disturbances. These findings provide important insights into the diagnosis and treatment of diabetes complicated by PWS.

## 1. Introduction

Prader–Willi syndrome (PWS) is a complex genetic disorder that affects multiple systems and results from the absence of expression of genes in the paternal chromosome 15q11.2–q13 region, with an incidence ranging from 1/10,000 to 1/30,000.^[1]^ Its main clinical manifestations include bulimia, obesity, short stature, developmental delay, neonatal hypotonia, and multiple endocrine disorders such as hypogonadism, growth hormone (GH)/insulin-like growth factor-I axis dysfunction, hypothyroidism, and central adrenal cortical insufficiency. Additional characteristics include scoliosis, intellectual disability, and behavioral and psychiatric disorders.^[2]^

The clinical features of PWS vary by age. During the fetal and neonatal periods, decreased fetal movement, hypotonia, weak crying, and feeding difficulties are the predominant signs. In contrast, obesity, gonadal dysplasia, and learning problems become the primary manifestations during adolescence.^[3]^ With advances in medical care, mortality in infancy and early childhood has declined, resulting in an increasing number of adolescents and adults with PWS. At this stage, morbid obesity becomes a defining feature, significantly increasing the risk of diabetes mellitus and cardiovascular disease, and has emerged as the major contributor to poor prognosis.^[4]^ Therefore, early and continuous monitoring of glucose metabolism status, beginning in adolescence or earlier, may play an important role in improving long-term outcomes.

Here, we present the case of an adult patient with PWS and diabetes mellitus and discuss the diagnostic and therapeutic considerations based on existing literature. Through this case, we aimed to enhance the clinician’s understanding of these conditions and emphasize the importance of early attention to glucose metabolism, thereby providing insights that may guide diagnosis and management.

## 2. Case description

A 20-year-old woman presented to our department with a 6-year history of elevated blood glucose levels. Six years earlier, she had been diagnosed with “diabetes mellitus” at a local hospital after elevated blood sugar levels were detected. She was treated with detemir insulin (32 units daily) and insulin aspart (12 units before each meal) combined with metformin (1500 mg/day). Despite therapy, glycemic control remained inadequate, even after the addition of dapagliflozin (10 mg/day) and acarbose (150 mg/day). Her fasting blood sugar levels were consistently maintained at 12.0 to 14.0 mmol/L, and her postprandial blood sugar remained at 17.0 to 22.0 mmol/L. She did not regularly adhere to dietary restrictions or exercise recommendations.

Regarding personal history, the patient was delivered via cesarean section at full term owing to an umbilical cord wrapped around the neck with 1 week of intrauterine hypoxia. At birth, she exhibited low muscle tone, absence of crying, and difficulty sucking, and no definitive diagnosis was made at the local hospital. At approximately 1 year of age, she could ambulate while using a wall for support. Beginning at 2 years old, she developed excessive eating owing to a strong appetite and overeating, which resulted in progressive weight gain. She had an intellectual disability and did not attend school. During puberty, she experienced no significant height increase. Her breasts did not develop, and she had no pubic hair or menstruation. Her parents and 12-year-old brother had normal body types and intelligence levels. Her parents were not consanguineous, and they denied a family history of genetic diseases.

Physical examination revealed a height of 150 cm, weight of 65 kg, and body mass index of 28.9 kg/m^2^. She had a narrow forehead, almond-shaped eyes, a low nasal bridge, full-moon facies, a thin upper lip, and short hands and feet. Her intelligence and cognitive functions were markedly lower than those of her peers. Hearing and olfactory functions were roughly assessed as normal. Cardiopulmonary and abdominal examinations showed no abnormalities.

## 3. Diagnostic assessment

The results of laboratory examinations performed after admission are presented in Table 1. Her fasting plasma glucose level was 21.51 mmol/L, and her hemoglobin A1c (HbA1c) was 12.5%. Triglyceride levels were 4.86 mmol/L. The oral glucose tolerance test and pancreatic β-cell function assessment, performed after fasting plasma glucose had stabilized (approximately 10.0 mmol/L), revealed severely impaired insulin secretion. Insulin autoantibody and glutamic acid decarboxylase antibodies were negative. Although her blood cortisol levels were low, her diurnal rhythm remained normal.

**Table 1:** The results of laboratory examinations performed after admission.

Laboratory	Results	Reference range
Glucose (mmol/L)	21.51	3.90–6.10
**Oral glucose tolerance test**		
Fasting glucose (mmol/L)	11. 5	
2-h glucose (mmoL/L)	22. 16	
**C-peptide (ng/mL**)		
C-peptide 0 min	0. 74	1.10–3.30
C-peptide 120 min	2.09	
Hemoglobin A1c	12.5%	4.0–6.0
Glycated albumin	27.6%	11–17
**Diabetes related autoantibodies**		
Insulin autoantibody (IU/mL)	4.804	0–20
Glutamic acid decarboxylase antibody (IU/mL)	5.662	0–30
Total cholesterol (mmol/L)	4.83	1.80–5.17
Triglyceride (mmol/L)	4.86	0.56–1.70
High density lipoprotein-cholesterol (mmol/L)	0.60	1.04–1.70
Low density lipoprotein-cholesterol (mmol/L)	2.23	0.45–3.15
Apolipoprotein A1 (g/L)	0.96	1.00–1.60
Apolipoprotein B (g/L)	1.28	0.60–1.10
**Hormonal exams**		
Luteinizing hormone (mUI/mL)	3.60	
Follicle-Stimulating hormone (mUI/mL)	8.40	
Estradiol(pmol/L)	134.30	
Progesterone (nmol/L)	0.83	
Prolactin(uIU/mL)	175.6	
Testosterone (nmol/L)	1.01	
Insulin-Like Growth Factor-1 (Before exercising)(ng/mL)	95.56	60.0–350.0
Growth hormone (Before exercising) (ng/mL)	0.754	0.06–5.00
**Adrenocorticotropic hormone (pg/mL**)		
8:00	13.85	6.0–40.0
**Cortisol (nmol/ L**)		
8:00	279.79	286.94–728.38
14:00	111.31	194.8–432.08
Free thyroxine (pmol/L)	14.34	11.50–22.70
Free Triiodothyronine (pmol/L)	4.72	3.50–6.50
Thyroid-stimulating hormone (uIU/mL)	2.647	0.55–4.78
Antithyroglobulin antibodies (IU/mL)	<15.0	0–60
Antithyroid peroxidase antibodies (IU/mL)	<28.0	0–60

Imaging examinations showed no significant abnormalities on pituitary magnetic resonance imaging. Bone age radiography demonstrated complete closure of the epiphyses. Pelvic magnetic resonance imaging revealed an underdeveloped uterus measuring approximately 15 × 64 × 26 mm^3^. Electromyography indicated bilateral sural nerve injury. Cytogenetic testing confirmed a 46, XX karyotype.

Whole exome sequencing identified an approximately 4.9 Mb heterozygous deletion in the chr15q11.2–q13.1 region (GRCh37:23606317–28544712) of the patient’s genomic DNA. Methylation-specific Multiplex Ligation-dependent Probe Amplification (MS-MLPA) technology further supported the diagnosis of PWS (Table 2). Additionally, sequencing analysis of the patient’s parents and younger brother was conducted. The mother and younger brother carried no mutations in the relevant region, whereas the father had a heterozygous mutation of uncertain significance (Table 3).

**Table 2:** The methylation analysis results of the patient.

Gene(Reference sequence)	Region	Copy number result	Methylation result	Conclusion
SNRPN (NM-022807.5)	15q11.2-q13	Deletion of CNV signal	The methylation level is approximately 100%.	PWS

CNV = copy number variation, PWS = Prader-Willi syndrome.

**Table 3:** Sanger sequencing results of the proband, her younger brother, and her parents.

Verified locus information	Relationship	Verification result
KMT2C chr7:151848652 Exon50 NM_170606.3:c.12541A > G (p.Lys4181Glu)	Proband	Heterozygous
	Younger brother	Wild-type
	Mother	Wild-type
	Father	Heterozygous

## 4. Therapeutic intervention, follow-up, and outcomes

The main treatment goals for this patient were to lower blood glucose levels, reduce weight, and provide symptomatic management. Insulin glargine (42 units before bedtime) and insulin aspart 15 units administered subcutaneously immediately before each meal (total 45 units/day), in combination with metformin (1500 mg/day), dulaglutide (0.75 mg/week, later increased to 1.5 mg/week after 2 weeks without discomfort), empagliflozin (10 mg/day), and chiglitazar sodium (48 mg/day), were administered to control blood glucose. Mecobalamin (1.5 mg/day) was prescribed for neural support. Fenofibrate (200 mg/day) was used to reduce triglyceride levels, and strict dietary and exercise guidance was provided to slow the development of obesity-related complications.

One month after discharge, the patient experienced a 1-kg weight loss, and her random blood glucose level decreased to 6.7 mmol/L, indicating adequate glycemic control at that time. However, during the subsequent year of follow-up, her blood glucose levels gradually increased due to persistent secretive food-seeking behavior and inability to regulate excessive eating. Her FBG ranged from 13.0 to 16.0 mmol/L, and her 2-h postprandial glucose ranged from 15.0 to 24.0 mmol/L, with her HbA1c worsening to 14.1%. No significant weight loss was observed during this period. She also had repeated episodes of urinary ketonuria; therefore, empagliflozin was discontinued, whereas insulin, metformin, chiglitazar sodium, and dulaglutide were continued. Despite this regimen, her glycemic control remained poor, with HbA1c levels exceeding 12.0%.

Two months prior, we modified her glucose-lowering regimen by replacing dulaglutide with tirzepatide (7.5 mg/week) and continuing insulin, metformin, and chiglitazar sodium. After 2 months of tirzepatide treatment, her weight decreased by 3 kg, her fasting blood sugar stabilized between 7.0 and 9.0 mmol/L, and her 2-h postprandial glucose ranged from 11.0 to 13.0 mmol/L. Her appetite was also mildly reduced compared to her previous state. Unfortunately, the 2‑month follow‑up HbA1c level was unavailable, owing to difficulties reported by the patient’s parents regarding transportation, adherence to scheduled appointments, and the patient’s lack of cooperation. Nevertheless, we continue to follow-up this patient.

## 5. Ethics approval and consent to participate

The study was approved by the Medical Ethics Committee of The Second Affiliated Hospital of Harbin Medical University(Approval No.KY2025-357). The patient and her kin have provided informed consent for publication of the case.

## 6. Discussion

PWS is caused by dysfunction or absence of an imprinted gene within the chromosome 15 region located at 15q11.2–15q13 on the paternal chromosome.^[5]^ The main genetic subtypes of PWS include paternal deletions (type I, type II, or atypical deletions of varying lengths depending on the proximal chromosomal breakpoint), maternal uniparental disomy, rare imprinting defects, and several very rare chromosomal rearrangements such as translocations involving the PWS region. An abnormal methylation profile of the SNRPN gene provides strong diagnostic support for PWS.^[6]^ The characteristic clinical features of PWS include hypotonia and feeding difficulties during infancy. As individuals age, excessive eating, food-seeking behavior, and ultimately morbid obesity become prominent, together with developmental delay, short stature, cognitive impairment, hypogonadism, and unique behavioral patterns such as temper tantrums, stubbornness, manipulative tendencies, and compulsive behaviors.^[7]^ For patients with suspected PWS in clinical practice, DNA methylation-specific polymerase chain reaction and MS-MLPA are recommended as the first-line diagnostic modalities, demonstrating a diagnostic sensitivity of over 99%.^[8]^ In this case, the patient exhibited the typical progression of PWS, and MS-MLPA analysis confirmed the diagnosis. However, the patient was not properly identified or diagnosed during infancy and childhood, and secondary diabetes was only diagnosed during adolescence when glycemic control was already poor, resulting in the loss of the optimal therapeutic window.

PWS is strongly associated with hyperphagia and life-threatening morbid obesity and is considered the most common cause of symptomatic obesity among known genetic disorders. Owing to chronic overeating and obesity, the incidence of diabetes in patients with PWS has increased, with a reported prevalence of 7% to 24%.^[9,10]^ Although patients with PWS are often severely obese, they may still exhibit relatively low circulating insulin levels,^[9]^ which was also observed in this case. Currently, no specific treatment guidelines exist for diabetes mellitus in PWS. Available pharmacologic options include metformin, α-glucosidase inhibitors, dipeptidyl peptidase-4 inhibitors, sodium-glucose cotransporter-2 (SGLT-2) inhibitors, and glucagon-like peptide-1 receptor agonists (GLP-1RAs). Metformin remains the first-line therapy and may reduce food-related anxiety and enhance satiety in some patients with PWS by improving insulin sensitivity.^[11]^ Studies have demonstrated that GLP-1RAs (including exenatide, liraglutide, and dulaglutide) can reduce ghrelin secretion, enhance satiety, aid in glycemic control, promote weight loss, and improve pre-meal insulin secretion in PWS.^[12,13]^ However, caution is advised owing to potential side effects such as delayed gastric emptying and the already existing risk of gastric rupture.^[14]^

In recent years, SGLT-2 inhibitors have received increased attention for their strong glucose-lowering and weight-reducing effects. Case reports suggest that combining GLP-1RAs with SGLT-2 inhibitors in PWS can lead to significant reductions in body weight and HbA1c, although dietary intake may remain unchanged.^[15]^ In this patient, supplementary exogenous insulin was required due to insufficient endogenous insulin secretion. Persistent overeating, lack of physical activity, reduced insulin sensitivity, and poor glycemic management warranted addition of metformin, a GLP-1RA, and an SGLT-2 inhibitor to insulin therapy. Furthermore, chiglitazar sodium, a novel peroxisome proliferator-activated receptor pan-agonist (α, γ, and δ), was added to improve metabolic dysfunction through multiple mechanisms, including glucose reduction, improved insulin sensitivity, lipid regulation, anti-inflammatory effects, and anti-fibrotic activity.^[16,17]^ This therapeutic combination enhanced appetite suppression, improved insulin sensitivity, supported weight reduction, and lowered blood glucose levels, contributing to better glycemic control.

Controlling obesity remains the central goal in managing PWS. Given that hyperphagia, impaired satiety, and associated eating behaviors are intrinsic to PWS, weight and glycemic management are particularly challenging. Tirzepatide, a dual agonist of the glucose-dependent insulinotropic polypeptide and glucagon-like peptide-1 (GLP-1) receptors, exerts its glucose-lowering effect by simultaneously improving β-cell function, insulin sensitivity, and α‐cell function in individuals with type 2 diabetes mellitus.^[18]^ In patients with inadequately controlled type 2 diabetes mellitus receiving basal insulin, tirzepatide, whether alone or in combination with other oral agents, has been shown to improve glycemic control without increasing the risk of hypoglycemia.^[19]^ Compared with traditional GLP-1RAs, tirzepatide offers greater reductions in body weight, HbA1c, and lipid parameters.^[20]^ Additionally, among individuals with obesity but without diabetes, tirzepatide has demonstrated superior reductions in body weight and waist circumference compared with other anti-obesity medications such as semaglutide.^[21,22]^ In this patient, although glycemic control initially improved with conventional medications, both weight and glucose levels remained suboptimal over time. Given the potent glucose-lowering and weight-reducing effects of tirzepatide, dulaglutide was replaced with tirzepatide. After 2 months of treatment, the patient experienced a significant 3-kg weight loss, improved blood glucose control, and mild appetite reduction, suggesting that tirzepatide may offer greater benefit for managing both glycemia and weight in PWS. Unfortunately, the 2‑month post‑treatment HbA1c level was not obtainable, as the patient failed to attend the scheduled follow‑up at our center: a well‑recognized challenge in PWS, where intellectual disability, behavioral issues, and limited caregiver support frequently undermine treatment and follow-up adherence. This represents a clear limitation of the present case report. Nevertheless, this experience offers a valuable real‑world insight: for PWS patients, evaluation of novel therapies should extend beyond biochemical efficacy to encompass feasibility, adherence, caregiver burden, and patient cooperation. In this regard, the once‑weekly subcutaneous dosing of tirzepatide may theoretically enhance adherence: an advantage that warrants exploration in future dedicated studies. In addition, the long-term efficacy and safety of tirzepatide in PWS remain uncertain and require continued monitoring and further research.

Despite pharmacologic therapy, the patient’s dietary intake remained largely unchanged. Her compulsive food-seeking behavior and inadequate parental supervision contributed to poor glycemic control. For patients with PWS, caregivers must enforce strict dietary restrictions, provide constant supervision, and encourage regular physical activity to slow the progression of obesity. Furthermore, previous studies have shown that GH therapy may improve body composition, basal energy expenditure, muscle strength, exercise tolerance, and fat-free mass in PWS.^[10,23,24]^ However, GH has antagonistic effects on insulin, necessitating careful evaluation of glucose and insulin levels before and after initiating GH treatment. In this case, GH therapy was not administered because of the patient’s low insulin levels, severe hyperglycemia, closed epiphyses, and parental refusal.

Most individuals with PWS experience hypogonadism, and this patient also exhibited hypogonadotropic hypogonadism. To induce, promote, or maintain puberty, sex hormone therapy is often required. Hormone replacement therapy may also enhance muscle mass and support normal bone formation. However, it remains controversial because androgen therapy in males may exacerbate behavioral issues, whereas estrogen therapy in females may lead to menstrual hygiene difficulties. Thus, hormone replacement therapy should be discussed thoroughly with guardians and initiated only with their full consent.^[25]^ In this case, the patient’s guardians declined sex hormone therapy despite counseling.

In summary, we report the case of an adult patient with classic manifestations of PWS. Owing to delayed diagnosis during infancy, the optimal therapeutic window was missed, and the patient later developed refractory diabetes mellitus. This case provides valuable insights, underscoring the importance of early recognition of PWS and the need for clinicians to consider genetic syndromes when encountering early-onset diabetes with distinctive phenotypic features. Effective management of PWS-associated diabetes requires a comprehensive approach integrating strict dietary supervision, behavioral interventions, and individualized pharmacotherapy. Emerging agents such as tirzepatide show promise for addressing core metabolic abnormalities in PWS; however, their long-term safety and efficacy warrant further investigation.

## Acknowledgments

The authors are very grateful to the patient for providing her case. We would like to thank Editage (www.editage.cn) for English language editing.

## Author contributions

**Data curation:** Junjie Yu.

**Supervision:** Jilin Wei, Meihua Liang.

**Writing – original draft:** Shuang Li.
